# Ketamine Administration Reverses Corticosterone-Induced Alterations in Excitatory and Inhibitory Transmission in the Rat Dorsal Raphe Nucleus

**DOI:** 10.1155/2019/3219490

**Published:** 2019-08-15

**Authors:** Joanna Sowa, Magdalena Kusek, Bartosz Bobula, Grzegorz Hess, Krzysztof Tokarski

**Affiliations:** Department of Physiology, Institute of Pharmacology, Polish Academy of Sciences, 12 Smetna Street, 31-343, Krakow, Poland

## Abstract

Ketamine, a N-methyl-D-aspartate (NMDA) receptor antagonist, exerts rapid antidepressant effects in human patients and ameliorates depressive-like behavioral effects of chronic stress in animal models. Chronic stress and elevated corticosterone levels have been shown to modify serotonin (5-HT) neurotransmission, and ketamine's antidepressant-like activity involves a 5-HT-dependent mechanism. However, it is not known if and how ketamine affects the electrophysiological characteristics of neurons and synaptic transmission within the dorsal raphe nucleus (DRN), the main source of 5-HT forebrain projections. Our study was aimed at investigating the effects of a single ketamine administration on excitatory and inhibitory transmission in the DRN of rats which had previously been administered corticosterone twice daily for 7 days. Spontaneous excitatory and inhibitory postsynaptic currents (sEPSCs and sIPSCs) were then recorded from DRN projection cells in ex vivo slice preparations obtained 24 h after ketamine injection. Repeated corticosterone administration increased sEPSC frequency and decreased sIPSC frequency in DRN projection cells. There were no changes either in the amplitude of postsynaptic currents or in the excitability of these cells. In slices prepared from rats with ketamine administered after the end of corticosterone treatment, the frequencies of sEPSCs and sIPSCs were similar to those in control preparations. These data indicate that a single administration of ketamine reversed the effects of corticosterone on excitatory and inhibitory transmission in the DRN.

## 1. Introduction

It has been well documented that administering ketamine, a N-methyl-D-aspartate (NMDA) receptor antagonist, to patients suffering from major depressive disorder and bipolar depression induces rapid antidepressant effects ([1] reviewed in [2, 3, 4]). Risk factors for depressive disorders include chronic stress and elevated cortisol levels (reviewed in [5, 6]). Studies in animal models have demonstrated that ketamine administration may ameliorate chronic stress-associated depressive-like behavioral changes along with stress-related morphological changes of nerve cells in the brain ([7] reviewed in [8, 9]). Antidepressant actions of ketamine are currently thought to be related to a rapid, transient increase in the extracellular glutamate level in the medial prefrontal cortex (mPFC). This leads to activity-dependent release of the brain-derived neurotrophic factor (BDNF), followed by the activation of tyrosine receptor kinase B (TrkB) receptors and mammalian target of rapamycin- (mTOR-) dependent intracellular signaling cascades. These mechanisms promote the strengthening of excitatory synapses in the mPFC and in subcortical reward circuits ([10] reviewed in [11, 12, 13]).

Moreover, studies in rats have shown that ketamine's antidepressant-like activity may involve a serotonin- (5-hydroxytryptamine- (5-HT-)) dependent mechanism. Depletion of 5-HT reduces antidepressant-like effects of ketamine in naïve rats when administered 24 h, but not 1 h, prior to behavioral testing [14]. Systemic ketamine administration increases the extracellular 5-HT level in the mPFC, and ketamine's antidepressant-like effects require activation of AMPA receptors in the dorsal raphe nucleus (DRN) [15]. DRN is the main source of long-range 5-HT projections, which regulate the activity of target forebrain structures, including the mPFC [16]. DRN projection neurons are controlled by several excitatory and inhibitory synaptic inputs. The mPFC is also a prominent source of glutamatergic innervation of the DRN, but glutamatergic inputs to the DRN also originate from subcortical structures including the lateral habenula, hypothalamus, periaqueductal gray, parabrachial nucleus, amygdala, and substantia nigra [17, 18]. These excitatory inputs form synapses both on DRN 5-HT projection neurons and local GABAergic interneurons, which inhibit projection neuron activity [18, 19]. The DRN also receives GABAergic inputs from certain subcortical regions (reviewed in [20]). Assessment of the balance between excitatory and inhibitory inputs to DRN projection cells is complicated by the fact that within the DRN, a fraction of GABA-containing terminals is organized in triads with glutamatergic terminals and a common postsynaptic target, and thus, GABA may presynaptically gate glutamate release [21]. A subpopulation of DRN glutamatergic neurons has been described in the literature, and many DRN 5-HT neurons coexpress markers for glutamate [17, 22, 23].

Repeated corticosterone administration is considered an animal model of stress-induced depressive-like behavior in rodents ([24, 25] reviewed in [26]). We have recently demonstrated that repeated corticosterone administration weakened GABAergic inputs to rat DRN projection neurons by decreasing the frequency of spontaneous inhibitory postsynaptic currents (sIPSCs) [27]. This effect is likely to contribute to the corticosterone treatment-induced dysregulation of the 5-HT system [28]. We have also demonstrated that treatment with a 5-HT_7_ receptor antagonist, which has fast antidepressant-like effects (reviewed in [29]), normalized the frequency of sIPSCs in the DRN of corticosterone-treated rats [27]. Since ketamine is known to reduce the depressive-like behavioral symptoms in repeated corticosterone-treated and stressed rats [7, 30], we investigated whether ketamine administration could reverse the changes in DRN excitatory and inhibitory synaptic transmission resulting from 7-day corticosterone treatment.

## 2. Materials and Methods

### 2.1. Animals

Male Wistar rats (Charles River, Germany) weighing approx. 150-160 g at the beginning of the experiment were housed in groups of 5 animals, under controlled 12 h light/dark cycle (light on: 07:00–19:00). Standard food and tap water were available *ad libitum*. The experimental procedures were approved by the Local Ethics Committee for Animal Experiments at the Institute of Pharmacology, Polish Academy of Sciences, and were carried out in accordance with the European Community guidelines for the use of experimental animals and national law. All efforts were made to minimize animal suffering and the number of animals used.

### 2.2. Treatment

The rats were assigned to four groups. Corticosterone (Sigma-Aldrich, suspended in 1% Tween 80) was injected subcutaneously (dose: 10 mg/kg, volume: 1 ml/kg), twice daily for 7 consecutive days. In the first experimental group (termed: Cort+Ket), animals received an injection of ketamine (Tocris; dose: 40 mg/kg, volume: 1 ml/kg, dissolved in 0.9% NaCl, subcutaneously), on the day after the end of corticosterone treatment. The ketamine dose was based on a study which demonstrated that ketamine reversed chronic unpredictable stress-induced depression-like behavior [7]. The second experimental group (termed: Cort+NaCl) received corticosterone for 7 days and, on the day after the end of corticosterone treatment, an injection of 0.9% NaCl. Control animals for these treatments received 1% Tween 80 for 7 days and either an injection of ketamine on the 8^th^ day (termed: Tween + Ket) or a single injection of 0.9% NaCl on the 8^th^ day (termed: Tween + NaCl). There were 10 rats in each group.

### 2.3. Slice Preparation and Whole-Cell Recording

Brain slices were prepared 24 h after the last substance administration to avoid acute effects of corticosterone and ketamine. Rats were anesthetized with isoflurane (Aerrane, Baxter, UK) and decapitated. Brains were quickly removed and placed in an ice-cold artificial cerebrospinal fluid (ACSF) containing (in mM) the following: 130 NaCl, 5 KCl, 2.5 CaCl_2_, 1.3 MgSO_4_, 1.25 KH_2_PO_4_, 26 NaHCO_3_, and 10 D-glucose, bubbled with the mixture of 95% O_2_ and 5% CO_2_. Coronal midbrain slices containing the DRN (thickness: 300 *μ*m) were cut using a vibrating microtome (Leica VT1000) and subsequently incubated in ACSF at 30 ± 0.5°C for at least 3 h. Two slices were obtained from each animal. Individual slices were placed in the recording chamber and superfused at 2.5 ml/min with warm (32 ± 0.5°C), modified ACSF of the following composition (in mM): 132 NaCl, 2 KCl, 2.5 CaCl_2_, 1.3 MgSO_4_, 1.25 KH_2_PO_4_, 26 NaHCO_3_, and 10 D-glucose, bubbled with 95% O_2_–5% CO_2_ [27].

Whole-cell recordings were obtained from the dorsal part of the midline region of the DRN. Neurons were visualized using the Zeiss Axioscope 2 upright microscope (Nomarski optics), a 40x water immersion lens, and an infrared camera. Recording pipettes were pulled from borosilicate glass capillaries (Harvard Apparatus), using the Sutter Instrument P97 puller. Pipettes had an open tip resistance of approx. 6 M*Ω*. The pipette solution contained (in mM) the following: 130 K-gluconate, 5 NaCl, 0.3 CaCl_2_, 2 MgCl_2_, 10 HEPES, 5 Na_2_-ATP, 0.4 Na-GTP, and 1 EGTA (osmolarity: 290 mOsm, pH = 7.2). Signals were recorded using the SEC 05LX amplifier (NPI, Germany), filtered at 2 kHz and digitized at 20 kHz using the Digidata 1440A interface and Clampex 10 software (Molecular Devices, USA) [27].

**Figure 1:**
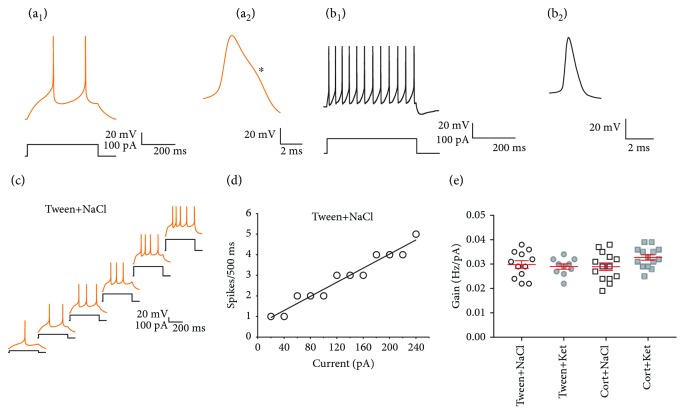
Repeated corticosterone and single ketamine injections do not influence basic membrane properties and excitability of DRN projection neurons. (a_1_) Response of a representative putative serotonergic neuron to a depolarizing current pulse and (a_2_) a single action potential of this cell at an extended timescale, with the “notch” on the descending phase marked with an asterisk. (b_1_) Response of a putative GABAergic interneuron to a depolarizing current pulse and (b_2_) a single action potential shown at an extended timescale. (c) Responses of a projection neuron to depolarizing current steps of increasing intensity (step: 20 pA; every second response and current pulse are shown) recorded in a DRN slice prepared from a control (Tween+NaCl) animal. (d) Relationship between spiking rate and injected current for the cell shown in (e). The slope of the linear regression line fitted to the experimental data represents the gain. (c) Summary graph showing mean gain (±SEM) of all neurons from the Tween+NaCl, Tween+Ket, Cort+NaCl, and Cort+Ket groups. The differences between groups are not significant.

Putative 5-HT DRN neurons were identified on the basis of their response to hyper- and depolarizing current pulses and a characteristic shape of the action potential (Figures 1(a_1_) and 1(a_2_)) [31]. After obtaining the whole-cell configuration and a subsequent 10 min stabilization period, the firing characteristics of the recorded cells were assessed using intracellular injections of rectangular current pulses of increasing amplitude (duration: 400 ms; Figure 1(c)) in the current clamp mode. For each cell, the relationship between the number of spikes and the injected current intensity was determined and the neuronal gain was determined as a slope of the linear regression line fitted to experimental data (Figures 1(d) and 1(e)).

**Figure 2:**
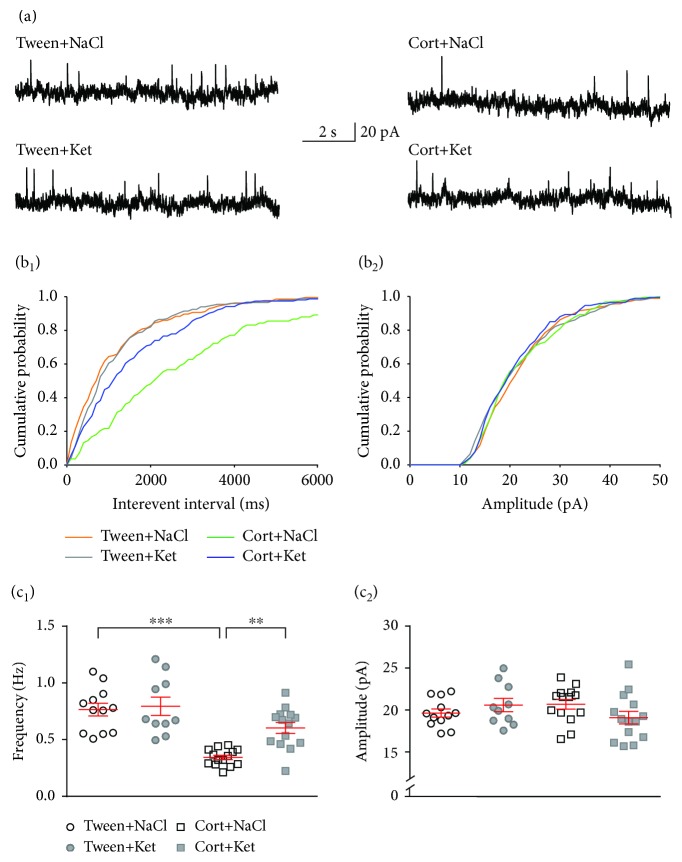
Single ketamine injection reverses the effect of repeated corticosterone administration on DRN glutamatergic transmission. (a) Sample recordings from representative neurons in slices prepared from animals treated with Tween+NaCl (*upper left trace*), Tween+Ket (*lower left trace*), Cort+NaCl (*upper right trace*), and Cort+Ket (*lower right trace*). (b_1_) Cumulative probability plots of interevent intervals of sEPSCs recorded from individual representative neurons from all four groups of rats. (b_2_) Cumulative probability plots of amplitudes of sEPSCs recorded from individual representative neurons. (c_1_) Summary graph showing the mean frequency (±SEM) of sEPSCs recorded from all neurons from the Tween+NaCl-, Tween+Ket-, Cort+NaCl-, and Cort+Ket-treated rats. ^∗∗∗^*p* < 0.001. (c_2_) Mean amplitudes (±SEM) of sEPSCs recorded from all neurons divided into the four investigated groups of animals (labels as in (c_1_)).

**Figure 3:**
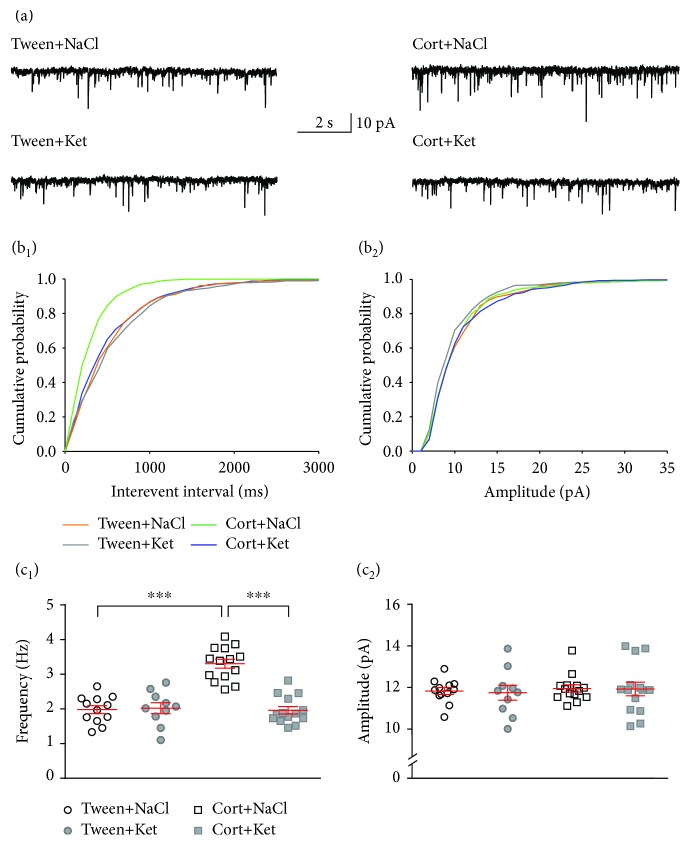
Single ketamine injection reverses the effect of repeated corticosterone administration on DRN GABAergic transmission. (a) Sample recordings from representative neurons in slices prepared from animals treated with Tween+NaCl (*upper left trace*), Tween+Ket (*lower left trace*), Cort+NaCl (*upper right trace*), and Cort+Ket (*lower right trace*). (b_1_) Cumulative probability plots of interevent intervals of sIPSCs recorded from individual representative neurons from all four groups of rats. (b_2_) Cumulative probability plots of amplitudes of sIPSCs recorded from individual representative neurons. (c_1_) Summary graph showing the mean frequency (±SEM) of sIPSCs recorded from all neurons from the Tween+NaCl-, Tween+Ket-, Cort+NaCl-, and Cort+Ket-treated rats. ^∗∗^*p* < 0.01 and ^∗∗∗^*p* < 0.001. (c_2_) A comparison of the mean amplitude (±SEM) of sIPSCs recorded from all neurons of the four investigated groups of animals (labels as in c_1_).

To record spontaneous excitatory postsynaptic currents (sEPSCs), cells were voltage-clamped at -76 mV and a 15 min stabilization period; synaptic events were recorded for 4 min as inward currents (Figure 2(a)). Next, cells were voltage-clamped at 0 mV, and after 15 min of stabilization, spontaneous inhibitory postsynaptic currents (sIPSCs) were recorded for 4 min as outward currents (Figure 3(a)) [31]. This approach allowed recording from the same neuron without needing to change the recording micropipette solution. We have previously shown that outward currents recorded from 5-HT neurons of DRN were completely blocked by bicuculline [31].

Spontaneous EPSCs and IPSCs were detected offline using Mini Analysis software (Synaptosoft), and individual synaptic events were selected manually for further analysis. Recordings were accepted for the analysis when the access resistance ranged between 15 and 18 M*Ω* and was stable (<25% change) throughout the recording. The threshold amplitude for EPSC detection was set to 6 pA and for IPSCs to 10 pA. EPSC and IPSC kinetics were determined from averaged EPSCs or IPSCs for each cell. The rise time was measured as the time needed for the current to rise from 10 to 90% of the peak. The decay time constant (tau) was determined from fitting an exponential function to the decay phase of the current [27].

### 2.4. Statistical Analysis

Rats were weighed on a daily basis. Average growth curves for each experimental treatment group were constructed by fitting linear regression lines to raw data. Slopes for the individual fits were compared across groups using the two-way ANOVA.

Statistical analysis of electrophysiological data was carried out using two-way ANOVA, followed by Tukey's multiple comparison test. The analysis was performed in GraphPad Prism 7 software. The results are expressed as the mean ± SEM. The significance level was set at *p* = 0.05 for all comparisons.

## 3. Results

### 3.1. Repeated Corticosterone Administration Affects Animal Body Weight

**Figure 4:**
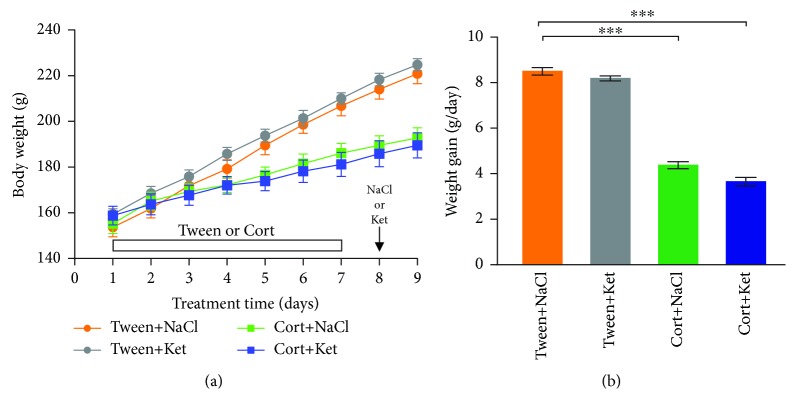
Effects of repeated corticosterone and single ketamine injections on animal body weight. Rats from the Cort+NaCl and Cort+Ket groups gained significantly less weight compared to control (Tween+NaCl) animals. No differences were evident between the Tween+NaCl and Tween+Ket groups. The number of animals in each group was 10. Shown are means ± SEM. ^∗∗∗^*p* < 0.001. The arrow indicates the day of either ketamine or NaCl injection.

Animals from the Cort+NaCl and Cort+Ket groups gained significantly less weight compared to rats receiving the vehicle (Tween+NaCl group; Figure 4(a)). No changes between the Tween+NaCl and Tween+Ket groups were evident (Figure 4(a)). A significant main effect of corticosterone on body weight gain was observed (*F*_(1, 36)_ = 759.5, *p* < 0.001). Rats from the Cort+NaCl and Cort+Ket groups gained weight significantly slower than Tween+NaCl-receiving animals (*p* < 0.001; Sidak's multiple comparison test; Figure 4(b)).

### 3.2. No Effect of Corticosterone and Ketamine, Alone and in Combination, on DRN Neuronal Excitability

All neurons subjected to analysis, when stimulated by depolarizing current pulses (400 ms), showed firing frequency adaptation with broad action potentials and a characteristic “notch” on their descending phase (Figures 1(a_1_) and 1(a_2_)). These features distinguish DRN projection neurons from local GABAergic interneurons (Figures 1(b_1_) and 1(b_2_)) (see also [32]). There were no statistically significant differences between any groups, either in the resting membrane potential or in the input resistance (Table 1). In addition, corticosterone and ketamine, alone and in combination, did not change the excitability of recorded DRN neurons (Figures 1(d) and 1(e), Table 1).

### 3.3. Ketamine Reverses the Effects of Corticosterone Treatment on the Excitatory Input to DRN Neurons

There was a significant effect of corticosterone treatment (*F*_(1, 46)_ = 24.96, *p* < 0.0001), ketamine treatment (*F*_(1, 46)_ = 26.57, *p* < 0.0001), and their interaction (*F*_(1, 46)_ = 30.14, *p* < 0.0001) on the frequency of sEPSCs. The sEPSC frequency was higher in the group receiving corticosterone and 0.9% NaCl injections (Cort+NaCl) compared to the Tween+NaCl group (3.30 ± 0.13 Hz vs. 1.98 ± 0.11 Hz, respectively; *n* = 14 and 12, *q* = 10.76, df = 46, *p* < 0.0001; Tukey's multiple comparison test), as well as when compared to the Cort+Ket group (3.30 ± 0.13 Hz vs. 1.96 ± 0.11 Hz, respectively; *n* = 14 and 14, *q* = 11.37, df = 46, *p* < 0.0001; Tukey's multiple comparison test; Figures 2(b_1_) and 2(c_1_)). No significant differences between the Cort+Ket and Tween+NaCl groups were observed (1.96 ± 0.11 Hz vs. 1.98 ± 0.11 Hz, respectively; *n* = 14 and 12, *q* = 0.1625, df = 46, *p* = 0.9994; Tukey's multiple comparison test). There were no significant effects of ketamine injection (Tween+Ket) on the sEPSC frequency compared to the control (Tween+NaCl) (2.02 ± 0.16 Hz vs. 1.98 ± 0.11 Hz, respectively; *n* = 10 and 12, *q* = 0.3159, df = 46, *p* = 0.9960; Tukey's multiple comparison test).

The analysis did not reveal any significant effects of treatment (corticosterone treatment: *F*_(1, 46)_ = 0.3357, *p* = 0.5651; ketamine treatment: *F*_(1, 46)_ = 0.03, *p* = 0.8632) or their interaction (*F*_(1, 46)_ = 0.0023, *p* = 0.9617) on the sEPSC amplitude (Figures 2(b_2_) and 2(c_2_)).

There were no effects of treatment with corticosterone (*F*_(1, 46)_ = 0.0312, *p* = 0.8605) or ketamine (*F*_(1, 46)_ = 0.0489, *p* = 0.8259) on the rise time of sEPSCs, and there was no interaction between factors (*F*_(1, 46)_ = 0.8218, *p* = 0.3694). The analysis did not reveal any effect of the treatments (corticosterone treatment: *F*_(1, 46)_ = 0.2944, *p* = 0.5900; ketamine treatment: *F*_(1, 46)_ = 0.0178, *p* = 0.8945) or their interaction (*F*_(1, 46)_ = 0.1061, *p* = 0.7461) on the decay time constant of sEPSCs (Table 2).

### 3.4. Ketamine Reverses the Effects of Corticosterone Treatment on the Inhibitory Input to DRN Neurons

There was a significant effect of corticosterone treatment (*F*_(1, 46)_ = 36.19, *p* < 0.0001), ketamine treatment (*F*_(1, 46)_ = 7.814, *p* = 0.0075), and their interaction (*F*_(1, 46)_ = 4.989, *p* = 0.0304) on the frequency of sIPSCs. The sIPSC frequency was lower in the group receiving corticosterone and 0.9% NaCl injections (Cort+NaCl) than in the Tween+NaCl group (0.34 ± 0.02 Hz vs. 0.77 ± 0.06 Hz, respectively; *n* = 14 and 12, *q* = 8.469, df = 46, *p* < 0.0001; Tukey's multiple comparison test) and the Cort+Ket group (0.34 ± 0.02 Hz vs. 0.60 ± 0.05 Hz, respectively; *n* = 14 and 14, *q* = 5.373, df = 46, *p* = 0.0023; Tukey's multiple comparison test; Figures 3(b_1_) and 3(c_1_)). No significant differences between the Cort+Ket and Tween+NaCl groups were observed (0.60 ± 0.05 Hz vs. 0.77 ± 0.06 Hz, respectively; *n* = 14 and 12, *q* = 3.306, df = 46, *p* = 0.1043; Tukey's multiple comparison test). There were no significant effects of ketamine injection (Tween+Ket) on the sIPSC frequency compared to the control (Tween+NaCl) (0.79 ± 0.08 Hz vs. 0.77 ± 0.06 Hz, respectively; *n* = 10 and 12, *q* = 0.5297, df = 46, *p* = 0.9819; Tukey's multiple comparison test).

The analysis did not reveal any effect of treatments (corticosterone treatment: *F*_(1, 46)_ = 0.1213, *p* = 0.7292; ketamine treatment: *F*_(1, 46)_ = 0.2282, *p* = 0.6351) or their interaction (*F*_(1, 46)_ = 3.673, *p* = 0.0615) on the sIPSC amplitude (Figures 3(b_2_) and 3(c_2_)).

There were no effects of treatment with corticosterone (*F*_(1, 46)_ = 1.796, *p* = 0.1867) or ketamine (*F*_(1, 46)_ = 0.4745, *p* = 0.4944) on the rise time of sIPSCs, and there was no interaction between factors (*F*_(1, 46)_ = 3.07, *p* = 0.0864). The analysis did not reveal any effect of treatment (corticosterone treatment: *F*_(1, 46)_ = 0.2679, *p* = 0.6072; ketamine treatment: *F*_(1, 46)_ = 0.0209, *p* = 0.8857) or their interaction (*F*_(1, 46)_ = 0.0228, *p* = 0.8805) on the decay time constant of sIPSCs (Table 3).

## 4. Discussion

We report that repeated injections of corticosterone increased sEPSC frequency and decreased sIPSC frequency recorded from putative DRN 5-HT projection neurons. This effect lasts for at least 2 days after the end of corticosterone treatment. There were no changes in the mean amplitude of sEPSCs or sIPSCs as well as no differences in their kinetics. This suggests a presynaptic mechanism of corticosterone-induced effects on glutamatergic and GABAergic transmission within the DRN. In slices prepared from rats which received an injection of ketamine one day after finishing corticosterone treatment, the frequencies of sEPSCs and sIPSCs recorded from DRN projection neurons were similar to those in control preparations. Thus, a single dose of ketamine reversed the effects of repeated corticosterone administration on spontaneous excitatory and inhibitory synaptic inputs to DRN projection neurons.

Rats from experimental groups receiving corticosterone gained weight significantly slower than control animals. This phenomenon is one of the correlates of depressive-like behavior in rats [26]. Acute administration of glucocorticoids is known to rapidly inhibit glutamatergic transmission to DRN 5-HT neurons by stimulating the retrograde endocannabinoid signaling pathway, which decreases presynaptic glutamate release [33]. However, in the case of repeated corticosterone administration, the mechanisms responsible for changes in synaptic transmission are not well understood. It has recently been shown that 21 consecutive daily injections of corticosterone (40 mg/kg) decreased sEPSC frequency in one subpopulation of DRN projection neurons while the sEPSC frequency in a different subgroup remained unchanged [34]. Our data indicate that shorter (7 days) treatment with a lower (10 mg/kg, twice daily) dose of corticosterone enhances glutamatergic input to DRN projection neurons. It is conceivable that the discrepancy between the two studies is because of different doses and durations of corticosterone treatment. Furthermore, the time of slice preparation was different in these two studies: 18 hours after the last injection of corticosterone [34] vs. 48 hours in our study. Since blood corticosterone levels in chronically treated animals remain elevated for at least 24 hours after the last injection [35], differences in experimental protocols might account for this discrepancy. No change in the probability of glutamate release has been reported in the DRN of rats subjected to 7-day chronic inescapable restraint stress [36]. We previously found that repeated injections of corticosterone lasting 7 days (10 mg/kg, twice daily) increased sEPSC frequency in the rat frontal cortex [37]. Interestingly, tonic endocannabinoid signaling has been implicated in the control of DRN glutamatergic transmission [38], and chronic stress reduces the ability of presynaptic CB1 receptors in the DRN to inhibit glutamate release [36]. Thus, it is conceivable that the observed increase in glutamatergic transmission in the DRN is due to impaired CB1 receptor function which would decrease the tonic inhibition of presynaptic glutamate release.

Our results show that repeated corticosterone treatment lasting for 7 days decreases the frequency of sIPSCs in rat DRN projection neurons. This is in agreement with our previous study in which an identical dose of corticosterone was administered twice daily for 14 days [27]. Reduction of the strength of the GABAergic input to a subset of DRN projection neurons involving a decrease in the frequency as well as the amplitude of sIPSCs has been reported to occur in mice subjected to 5-day social defeat stress [39]. It should be noted that CB1 receptors have also been implicated in the modulation of GABA release from local DRN interneurons [40], but it remains unknown whether stress or corticosterone modifies this aspect of CB1 receptor function.

The corticosterone-induced enhancement of the excitatory and reduction of inhibitory inputs to DRN projection neurons that we report in this study, combined with the corticosterone-induced reduction in the autoinhibitory function of 5-HT_1A_ somatodendritic receptors [25], are likely to increase the reactivity of DRN 5-HT cells. Increased concentrations of the 5-HT metabolite 5-hydroxyindoleacetic acid (5-HIAA) in the rat frontal cortex have been shown to occur after 12 days of corticosterone administration. This suggests higher 5-HT metabolism in the DRN [41]. In line with these findings, treatment with corticosterone elevates basal expression of tryptophan hydroxylase 2 (a rate-limiting enzyme for 5-HT synthesis; TPH2) in the DRN [28], as well as stress-induced increases in TPH2 activity in DRN neurons. These effects have been attributed to the increased expression of corticotropin-releasing hormone (CRH) and stronger activation of the CRH type 2 receptor (CRHR2) expressed on 5-HT neurons [42].

Changes in the frequency of sEPSCs and sIPSCs recorded from putative 5-HT projection neurons following corticosterone administration were reversed by a single dose of ketamine. This is in line with an earlier study reporting that 5-HT mediates ketamine's antidepressant-like effects in stressed animals exhibiting a depression-related phenotype [14]. Importantly, ketamine itself did not influence any of the parameters characterizing sEPSCs and sIPSCs recorded in animals that had not received corticosterone, consistent with the findings of other investigators [43]. It has been reported that acute effects of ketamine on 5-HT neurons in DRN slices include a transient, activity-independent increase in the frequency of AMPA receptor-mediated sEPSCs due to increased probability of spontaneous glutamate release [43]. In vivo, ketamine administration dose-dependently increases 5-HT levels in the mPFC. Infusion of AMPA receptor antagonists into the DRN blocks this effect [15, 44]. The mechanism underlying the antidepressant-like effect of ketamine may involve increased activity in the descending mPFC-DRN glutamatergic connections which physiologically activate DRN 5-HT projection cells [15]. Interestingly, it has been shown that activation of 5-HT DRN projection cells by ketamine is mediated by the cholinergic projection from the pedunculopontine tegmental nucleus [45]. However, these studies were performed using naïve animals, not subjected to stress or corticosterone administration. It should also be noted that the antidepressant effect of R-ketamine was reported to be independent of 5-HT [46]. We have used a racemic mixture of the two enantiomers, R- and S-ketamine. We demonstrate that ketamine administration reduces the strength of glutamatergic inputs to DRN projection neurons which are increased by corticosterone treatment and increases the strength of GABAergic inputs which are decreased by corticosterone. Together, these effects are likely to reduce the activity of DRN 5-HT projection cells altered by corticosterone. Thus, the mechanisms of ketamine-induced effects on DRN neurons might differ between naïve and corticosterone-treated animals. For example, the ketamine-related antidepressant action of 5-HT within the mPFC has been attributed to the activation of cortical 5-HT_1A_ receptors [47]. However, we have demonstrated previously that repeated corticosterone administration profoundly attenuated the electrophysiological effects of 5-HT_1A_ receptor activation in the frontal cortex [48]. This does not support the postulated involvement of 5-HT_1A_ receptors. The PFC-DRN projections have been found to mediate prophylactic effects of ketamine in preventing stress-related anxiety-like behaviors in mice [49], but further investigation is needed to uncover the mechanisms involved.

The effects of ketamine within the DRN do not appear to be limited to its blocking action on NMDA receptors. Other NMDA receptor antagonists do not increase sEPSC frequency in DRN projection neurons [43]. Ketamine has been demonstrated to increase phosphorylated mTOR levels in the DRN for at least 24 hours [43] after administration. Overactivation of mTOR signaling is known to enhance both glutamatergic and GABAergic transmission in striatal and hippocampal preparations by increasing the number of synaptic vesicles available for release, the number of synapses formed, and the miniature event size [50]. Thus, it is conceivable that the observed reversal of corticosterone-induced alterations in GABAergic transmission might be mediated by the mTOR signaling pathway. Further studies are therefore needed to conclusively identify the mechanisms of ketamine action on stress-related alterations in excitatory and inhibitory transmission in the DRN.

## 5. Conclusion

In conclusion, our results indicate that repeated administration of exogenous corticosterone strengthens excitatory and weakens inhibitory transmission within the DRN neuronal network. A single administration of ketamine reverses these lasting effects of elevated corticosterone levels within the DRN. These findings may be of importance for the development of novel treatment strategies to deal with stress-related disorders.

## Figures and Tables

**Table 1:** Effects of the treatments on the basic electrophysiological properties of recorded neurons (mean ± SEM).

Group	*V*_*m*_ (mV)	*R*_*m*_ (M*Ω*)	Gain (Hz/pA)	*n*
Tween+NaCl	−65.42 ± 1.45	596.40 ± 13.45	0.030 ± 0.002	12
Tween+Ket	−67.00 ± 1.73	583.50 ± 25.42	0.029 ± 0.001	10
Cort+NaCl	−64.14 ± 1.84	555.40 ± 29.78	0.029 ± 0.001	14
Cort+Ket	−66.43 ± 0.91	603.70 ± 17.18	0.033 ± 0.001	14

*V_m_*: resting membrane potential; *R_m_*: input resistance; *n*: number of cells. Differences between values are not significant (*p* > 0.05).

**Table 2:** Effects of the treatments on sEPSC characteristics (mean ± SEM).

Group	Mean frequency (Hz)	Mean amplitude (pA)	Rise time (ms)	Decay time constant (*τ*, ms)	*n*
Tween+NaCl	1.98 ± 0.11	11.81 ± 0.17	1.23 ± 0.04	5.44 ± 0.22	12
Tween+Ket	2.02 ± 0.16	11.75 ± 0.36	1.18 ± 0.04	5.40 ± 0.22	10
Cort+NaCl	3.30±0.13^∗∗∗^	11.95 ± 0.17	1.19 ± 0.03	5.27 ± 0.17	14
Cort+Ket	1.96 ± 0.11^###^	11.92 ± 0.33	1.22 ± 0.05	5.36 ± 0.20	14

*n*: number of cells. ^∗∗∗^*p* < 0.001 Tween+NaCl vs. Cort+NaCl; ^###^*p* < 0.001 Cort+NaCl vs. Cort+Ket; Tukey's multiple comparison test.

**Table 3:** Effects of the treatments on sIPSC characteristics (mean ± SEM).

Group	Mean frequency (Hz)	Mean amplitude (pA)	Rise time (ms)	Decay time constant (*τ*, ms)	*n*
Tween+NaCl	0.77 ± 0.06	19.64 ± 0.49	1.79 ± 0.14	6.56 ± 0.32	12
Tween+Ket	0.79 ± 0.08	20.60 ± 0.79	1.67 ± 0.13	6.47 ± 0.20	10
Cort+NaCl	0.34±0.02^∗∗∗^	20.69 ± 0.57	1.75 ± 0.08	6.37 ± 0.18	14
Cort+Ket	0.60 ± 0.05^##^	19.10 ± 0.75	2.04 ± 0.12	6.38 ± 0.32	14

*n*: number of cells. ^∗∗∗^*p* < 0.001 Tween+NaCl vs. Cort+NaCl; ^##^*p* < 0.01 Cort+NaCl vs. Cort+Ket; Tukey's multiple comparison test.

## Data Availability

The experimental data used to support the findings of this study are included within the article.
