# Erratum to “Ketamine Administration Reverses Corticosterone-Induced Alterations in Excitatory and Inhibitory Transmission in the Rat Dorsal Raphe Nucleus”

**DOI:** 10.1155/2020/1514094

**Published:** 2020-08-28

**Authors:** Joanna Sowa, Magdalena Kusek, Bartosz Bobula, Grzegorz Hess, Krzysztof Tokarski

**Affiliations:** Department of Physiology, Institute of Pharmacology, Polish Academy of Sciences, 12 Smetna Street, 31-343, Krakow, Poland

In the article titled “Ketamine administration reverses corticosterone-induced alterations in excitatory and inhibitory transmission in the rat dorsal raphe nucleus” [1], there was an error in Figures 2 and 3. Figures 2 and 3 images were swapped. These errors occurred during the production process. The correct figures are as follows.

## Figures and Tables

**Figure 1:**
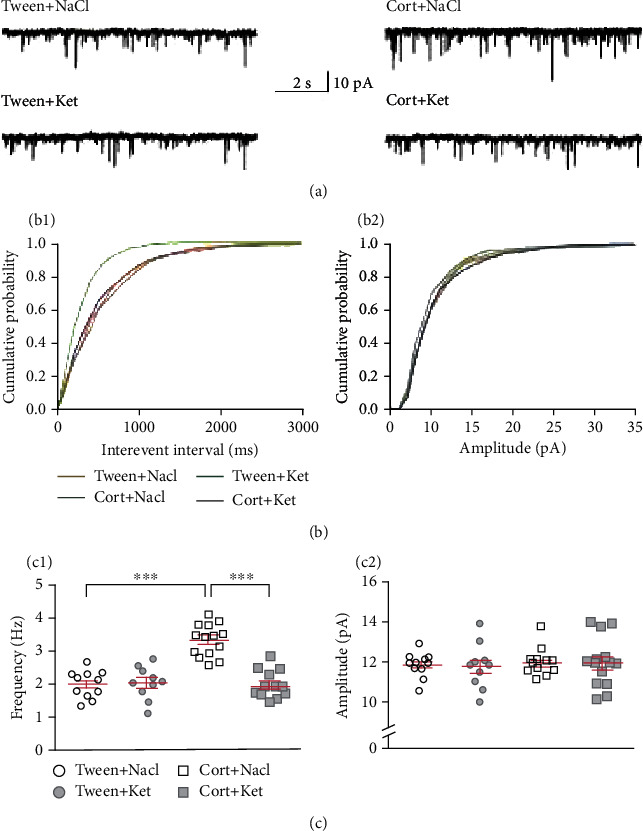
Single ketamine injection reverses the effect of repeated corticosterone administration on DRN glutamatergic transmission. (a) Sample recordings from representative neurons in slices prepared from animals treated with Tween+NaCl (upper left trace), Tween+Ket (lower left trace), Cort+NaCl (upper right trace), and Cort+Ket (lower right trace). (b_1_) Cumulative probability plots of interevent intervals of sEPSCs recorded from individual representative neurons from all four groups of rats. (b_2_) Cumulative probability plots of amplitudes of sEPSCs recorded from individual representative neurons. (c_1_) Summary graph showing the mean frequency (±SEM) of sEPSCs recorded from all neurons from the Tween+NaCl-, Tween+Ket-, Cort+NaCl-, and Cort+Ket-treated rats. ^∗∗∗^*p* < 0 001. (c_2_) Mean amplitudes (±SEM) of sEPSCs recorded from all neurons divided into the four investigated groups of animals (labels as in (c_1_)).

**Figure 2:**
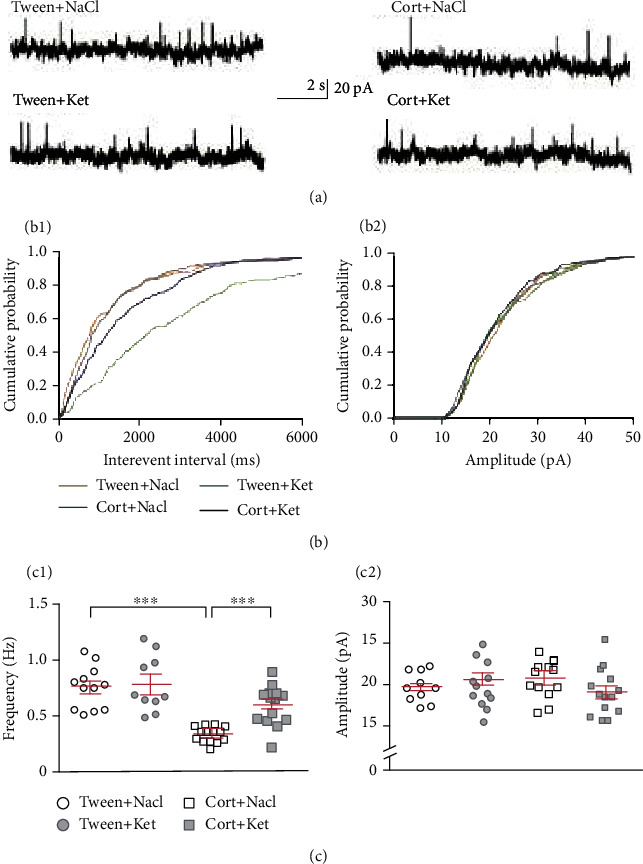
Single ketamine injection reverses the effect of repeated corticosterone administration on DRN GABAergic transmission. (a) Sample recordings from representative neurons in slices prepared from animals treated with Tween+NaCl (upper left trace), Tween+Ket (lower left trace), Cort+NaCl (upper right trace), and Cort+Ket (lower right trace). (b_1_) Cumulative probability plots of interevent intervals of sIPSCs recorded from individual representative neurons from all four groups of rats. (b_2_) Cumulative probability plots of amplitudes of sIPSCs recorded from individual representative neurons. (c_1_) Summary graph showing the mean frequency (±SEM) of sIPSCs recorded from all neurons from the Tween+NaCl-, Tween+Ket-, Cort+NaCl-, and Cort+Ket-treated rats. ^∗∗^*p* < 0 01 and ^∗∗∗^*p* < 0 001. (c_2_) A comparison of the mean amplitude (±SEM) of sIPSCs recorded from all neurons of the four investigated groups of animals (labels as in (c_1_)).
